# Inhibition of nitric oxide synthesis enhances leukocyte rolling and adhesion in human microvasculature

**DOI:** 10.1186/1476-9255-9-28

**Published:** 2012-07-19

**Authors:** Mokarram Hossain, Syed M Qadri, Lixin Liu

**Affiliations:** 1 Department of Pharmacology, College of Medicine, University of Saskatchewan, 107 Wiggins Road, Saskatoon, SK, S7N 5E5, Canada

**Keywords:** Leukocyte recruitment, nitric oxide, P-selectin, E-selectin, L-NAME, SCID-Hu

## Abstract

**Background:**

Nitric oxide (NO) is a multifunctional signaling molecule that regulates important cellular events in inflammation including leukocyte recruitment. Previous studies have shown that pharmacological inhibition of NO synthesis induces leukocyte recruitment in various in vitro and animal models. However, it is not known whether NO modulation has similar effects on leukocyte-endothelial cell interactions within the human microvasculature. The present study explored the effect of systemic L-NAME treatment on leukocyte recruitment in the SCID-hu mouse model.

**Methods:**

Human skin xenografts were transplanted in SCID mice to study human leukocyte dynamics in human vasculature. Early events of human leukocyte recruitment in human vasculature were studied using intravital microscopy. NO synthesis was pharmacologically inhibited using N^G^-nitro-L-arginine methyl ester (L-NAME). Immunohistochemical analysis was performed to elucidate E-selectin expression in human xenograft skin. Human neutrophil-endothelial cell interactions were also studied in an in vitro flow chamber assay system. P- and E-selectin expression on cultured human umbilical vein endothelial cells (HUVECs) was measured using ELISA. Platelet-activating factor (PAF) synthesis was detected using a TLC-based assay.

**Results:**

L-NAME treatment significantly enhanced the rolling and adhesion of human leukocytes to the human vasculature. Functional blocking of P- and E-selectins significantly inhibited rolling but not adhesion induced by inhibition of NO synthesis. Systemic L-NAME treatment enhanced E-selectin expression in human xenograft skin. L-NAME treatment significantly enhanced P- and E-selectin expression on HUVECs. L-NAME treatment did not significantly modify neutrophil rolling or adhesion to HUVECs indicating that L-NAME−induced subtle P- and E-selectin expression was insufficient to elicit dynamic neutrophil-HUVEC interactions in vitro. Moreover, synthesis of endothelial-derived PAF was not significantly modified by L-NAME treatment. These results point to the accelerated leukocyte recruitment in human vasculature following suppression of NO synthesis, effects that are mediated by P- and E-selectins. The findings are, however, not supported by the in vitro data.

**Conclusion:**

Inhibition of endogenous NO triggers early events of human leukocyte recruitment in human vasculature, involving complex cellular or molecular mechanisms in addition to P- and E-selectin-mediated leukocyte rolling.

## Background

Leukocyte recruitment is a dynamic cellular and molecular process in inflammation and constitutes leukocyte tethering, rolling, adhesion and ultimately emigration from the microvasculature [1-3]. The pleiotropic functions of the endogenous mediator nitric oxide (NO) include suppressing inflammatory responses such as leukocyte recruitment [4,5]. The mechanisms of leukocyte adhesion and emigration elicited by NO suppression are not completely understood [6]. The induction of NO through inducible NO synthase was previously shown to attenuate neutrophil chemotaxis [7-11]. N^G^-nitro-L-arginine methyl ester (L-NAME) inhibits the biosynthesis of NO from L-arginine by NO synthase. L-NAME pre-treatment was previously shown to increase leukocyte adhesion [12-14] and inhibit transendothelial migration of neutrophils [15]. Modulation of endogenous NO synthesis may further participate in F-actin depolymerization and expression of integrins [16].

NO has been shown to modulate the expression of selectins [17] which mediate tethering and rolling during leukocyte recruitment. L-NAME treatment may enhance the expression of endothelial adhesion molecules [13] such as P-selectin expression and enhance leukocyte rolling and adhesion [18]. NO inhibition may also stimulate degranulation of mast cells which release pro-inflammatory mediators that upregulate P-selectin expression [19,20]. Previous studies demonstrated that oxidant-induced P-selectin expression and leukocyte rolling were enhanced by the inhibition of endogenous NO [21]. However, the mechanism by which NO stimulates rapid mobilization of P-selectin fostering early leukocyte recruitment in vivo remains elusive.

The firm adhesion of leukocytes is mediated by platelet-activating factor (PAF) synthesis but it is unclear whether endogenous NO regulates the synthesis of this phospholipid. The role of NO in the modulation of E-selectin expression and function is not completely understood. In unstimulated endothelial cells, inhibition of NO synthesis for up to 4 h has no effect on E-selectin expression [22]. In IL-1α-stimulated endothelial cells, NO decreased E-selectin expression whereas other investigators did not observe the effects of NO on cytokine-induced E-selectin expression [6,23]. As expression and function are not always synonymous and since the functional assessment of selectins requires flow conditions, the potential effects of endogenous NO on the functions of P-selectin and E-selectin during leukocyte recruitment in human microvasculature require additional investigation.

Discrepancies exist in the exact role of NO in leukocyte recruitment due to the diversity of pharmacological approaches, tissues, and animal models studied. The present study explored the effect of NO modulation by L-NAME treatment on human leukocyte recruitment in human vasculature in the SCID-Hu mouse model and on the expression and function of selectins and PAF in vitro.

## Methods

### SCID-hu mouse model

CB-17 SCID mice (Harlan, Mississauga, ON), 6–8 weeks of age were grafted with partial thickness human skin according to a previously described protocol [24,25]. Animal protocols were approved by the local Animal Care Committee and met the guidelines of the Canadian Council for Animal Care. Briefly, partial thickness skin grafts from healthy human donors were prepared from discarded skin obtained from breast reduction surgeries using a 0.1-in. dermatome. A portion of the posterior thorax on the back (5 mm × 5 mm) was excised in halothane anesthetized recipient mice and human skin of the same size was placed and allowed to heal for 4 weeks before the mice were used for intravital microscopy. In the SCID-hu mouse model, transplanted human skin retains human dermal microvascular bed which connects with the mouse microvasculature as a result of spontaneous anastomosis [25].

### Isolation and labelling of human leukocytes

Twenty mL of heparinised blood collected from healthy volunteers was centrifuged (1350 rpm, 7 min, room temperature) and the buffy coat was collected and washed with HBSS containing Ca^2+^ and Mg^2+^ (Gibco, Grand Isle, NY). To study the interactions between human leukocytes and human vascular endothelium of human skin graft in SCID mice using fluorescent intravital microscopy, human leukocytes were labelled with rhodamine 6-G (0.025 % final concentration, 5 min; Sigma, St. Louis, MO). Unbound rhodamine 6-G was removed by washing three times in HBSS.

### Intravital microscopy

Mice were administered with L-NAME (50 mg/kg b.w., i.p.; Bachem, Torrance, CA) for 4 h before viewing the human skin microvasculature using intravital microscopy. Animals were anesthetised i.p. with ketamine (160 mg/kg b.w.) and xylazine (10 mg/kg b.w.) and body temperature was regulated using a heating pad [24]. Functional blocking antibodies and additional anesthetic was introduced through a cannulated jugular vein and infusion of rhodamine 6-G-labelled human leukocytes was through cannulated carotid artery. A flap consisting of the human skin graft was laid flat onto a pedestal, secured with suture, perfused with 37 °C-warmed saline and covered with a glass coverslip. The preparation was examined using an upright fluorescent microscope (Optiphot-2; Nikon) with a 20× water immersion objective. To identify human vessels, 100 mg of FITC-labelled *Ulex europaeus* (Sigma) was injected i.v. immediately before fluorescence microscopic visualization (excitation: 450–490 nm and emission: 520 nm). Rhodamine 6-G-labelled human leukocytes were visualized by excitation at 510–560 nm using a 590 nm emission filter. Images of the labelled human leukocytes and human microvessels were visualized using a silicon-intensified CCD camera (C-2400-08; Hamamatsu Photonics, Bridgewater, NJ) and recorded for playback analysis. Rolling of human leukocytes was expressed as percentage flux fraction, determined by counting the number of interacting human leukocytes in an individual vessel relative to the total number of human leukocytes passing through the vessel over the same period (determined by frame-by-frame analysis). Rhodamine 6-G-labelled human leukocytes that remained stationary on the vascular wall for at least 30 s were defined as adherent. Recording was started immediately after infusion of the labelled-leukocytes and the interactions were recorded for 30 min, a time point when the number of circulating labelled leukocytes was significantly diminished. Where indicated, the blocking mAbs (20–40 μg/mL) anti-human P-selectin G1 (BD Biosciences, Mississauga, ON) and/or anti-human E-selectin ES1 (kindly provided by Dr. KD Patel, University of Calgary, Calgary, AB), were injected i.v. as a bolus in a total volume of 100 μL of PBS after baseline interactions had been recorded. The antibodies were allowed to circulate for 2–3 min before a second bolus of human leukocytes was injected. Functional blocking is expected not to reverse leukocyte adhesion. Analysis of selectin functional blocking was thus, performed by determining the number of adherent leukocytes 5 min after the administration of blocking mAb.

### Immunohistochemical analysis

Human skin samples were harvested from representative SCID mice after intravital microscopy. These samples were then frozen in OCT and cut into 5-μm thick sections. Sections were stained with polyclonal goat anti-human E-selectin antibody to examine the level of E-selectin expression and then with biotin-conjugated secondary rabbit anti-goat antibody (Jackson ImmunoResearch Laboratories, Burlington, ON). Color was developed using the ABC kit (Vector Laboratories, Burlingam, CA) and chromagen diaminobenzadine (Sigma) and the sections were then counterstained with Gill II hematoxylin. Images were captured using a CCD digital camera (Nikon).

### Cell culture

Human umbilical vein endothelial cells (HUVECs) were harvested from fresh human umbilical cords and cultured as described previously [26]. After confluence was reached, the cultured HUVECs were trypsinzied for detachment and then seeded onto fibronectin-coated coverslips or 48-well plates. Since senescent endothelial cells express 50 % to 75 % less eNOS mRNA compared to their primary or first-passaged counterparts [27], HUVECs were, thus, used at first passage for all experiments.

### Isolation of human neutrophils

Human neutrophils were isolated from ACD (Acid Citrate Dextrose) anti-coagulated whole blood from healthy donors. After dextran (Spectrum Chemicals, Gardena, CA) sedimentation, isolation of neutrophils was performed at room temperature by using centrifugation through a density gradient of Ficoll Type 400 (Sigma) with 10 % Hypaque Sodium® (Sterling-Winthrop, Markham, ON). Isolated neutrophils were 97 % pure and 95 % viable. Purified human neutrophils were resuspended in HBSS with Ca^2+^ and Mg^2+^ at a concentration of 1×10^6^ cells/mL prior to use in laminar flow chamber assay.

### Laminar flow chamber assay

Coverslips with cultured HUVECs were mounted in a parallel plate flow chamber [28]. Reagents were added to the neutrophil suspension at the indicated time. The flow chamber was placed onto the stage of an inverted microscope (Carl Zeiss, Toronto, ON) and HUVEC monolayers were visualized at 100× magnification using phase contrast imagery with a 0.45 mm^2^ field of view. The stage area was enclosed in a warm air cabinet and maintained at 37°C prior to perfusion using a water bath. A syringe pump (Harvard Apparatus, Laval, QC) was used to draw the cells through the flow chamber at 2 dynes/cm^2^ for 20 min. Experiments were recorded via a CCD camera for analysis. Interacting cells were either rolling or adherent to the surface of the cover slip. A neutrophil that remained stationary for 10 sec or more was considered as adherent.

### Measurement of endothelial cell surface expression of P- and E-selectins

Surface P- and E-selectin expression on HUVECs was measured by ELISA. Cells were plated into a 48-well plate and used 24−48 h later. HUVEC monolayers were exposed to L-NAME or histamine (Sigma) for 10 min to induce P-selectin upregulation and with L-NAME or recombinant human TNFα (Collaborative Biochemical Products; Bedford, MA) for 4 h for E-selectin upregulation. After fixation (1 % formalin at 4 °C for 30 min) and rinsing with PBS, blocking was performed with 1 % BSA in PBS (30 min at 37 °C). The cells were incubated with the anti-P-selectin antibody S12 (2 μg/mL) or with the anti-E-selectin antibody EL246 (50 μg/mL) for 30 min at 37 °C, washed with 0.05 % Tween-20 followed by incubation (30 min at 37 °C) with the secondary antibody (peroxidase labelled goat-anti-mouse, 1:1000 dilution). Absorbance was measured at 450 nm after addition of TMB substrate (Dako Carpinteria, CA) and 0.01 H_2_SO_4_.

### PAF measurement

To determine the effect of L-NAME on PAF synthesis, a previously described method was used [29]. Briefly, HUVECs were incubated with L-NAME (100 μM) for 10 min at 37 °C in the presence of ^3^H-acetate. Histamine (10^-5^ or 10^-6^ M) or HBSS was then added to HUVECs and further incubated for 5 min at 37°C. The cells were then incubated with acidified methanol, scraped and added to a Bligh-Dyer extraction with cold PAF. Methanol was added until a monophase which was then split. After centrifugation (800–1000 rpm), the upper phase was removed and the lower phase was dried and spotted on a TLC plate and the resultant spots counted.

### Statistical analysis

Data are expressed as means ± SEM. As indicated in the figure legends, the statistical analysis in two value sets was made using paired or unpaired Student’s *t* test and the statistical differences among the multiple groups were analyzed by ANOVA and post hoc comparisons by the Tukey-Kramer Multiple Comparison Test. Values of p < 0.05 were considered statistically significant.

## Results

**Figure 1:**
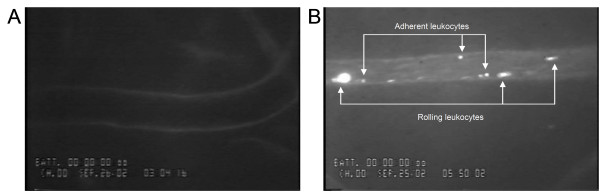
**Human leukocyte interactions with the human microvasculature. A**. Representative image of a FITC-conjugated *Ulex europaeus*-stained human post-capillary venule from the control SCID-hu mouse. **B**. Representative image of rhodamine 6-G labelled human leukocytes rolling and adhering to a *Ulex europaeus*-stained human post-capillary venule after 4-hour L-NAME treatment (50 mg/kg b.w., i.p.) in SCID-hu mice.

In the first series of experiments, we analyzed the in vivo effects of systemic L-NAME treatment (50 mg/kg b.w.) on human leukocyte recruitment in the human vasculature. Rhodamine 6-G-labelled human leukocytes were infused into SCID mice bearing human skin transplant and the interaction between the labelled human leukocytes and FITC-*Ulex europaeus* labelled-human microvasculature was visualized using fluorescence intravital microscopy (Figure 1).

**Figure 2:**
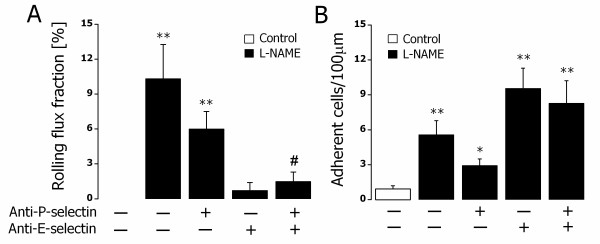
**L-NAME-elicited human leukocyte rolling and adhesion in vivo. A**. Leukocyte rolling flux fraction in untreated mice (control, white bar) and L-NAME (50 mg/kg b.w., i.p.)-treated mice (black bars). ** indicates P < 0.01 difference from untreated mice (*t* test). # indicates P < 0.05 difference from L-NAME-treated group in the absence of functional blocking antibodies (*t* test). + and – indicate the presence and absence of functional blocking anti-P- and/or anti-E-selectin antibodies, respectively. Data are means ± SEM (n = 2−6; number of vessels studied = 4−13; control: n = 4; number of vessels = 7). **B**. Leukocyte adhesion in untreated mice (control, white bar) or L-NAME (50 mg/kg b.w., i.p.)-treated mice (black bars). * and ** indicate P < 0.05 and P < 0.01 respectively from untreated mice (*t* test). + and – indicate the presence and absence of functional blocking anti-P- and/or anti-E-selectin antibodies, respectively. Data are means ± SEM (n = 3−8; number of vessels studied = 4−16; control: n = 4; number of vessels = 8).

Systemic L-NAME treatment significantly enhanced the rolling and adhesion of labeled leukocytes to the human vasculature. The involvement of P- and E-selectins in this process was determined utilizing two blocking antibodies directed against surface-expressed P- and E-selectins. As shown in Figure 2A, in the absence of L-NAME treatment (control), the rolling flux fraction was virtually undetectable as the number of labeled human leukocytes constitutes a relatively low proportion of the total circulating leukocytes despite baseline E-selectin expression (n = 4; number of vessels = 7). L-NAME treatment significantly enhanced the rolling flux fraction and blocking either P-selectin (n = 3; number of vessels = 9) or E-selectin (n = 2; number of vessels = 4) tended to decrease this process. Blocking both P- and E-selectins, however, significantly blunted the increased rolling flux fraction induced by L-NAME treatment (P < 0.05). Furthermore, L-NAME treatment significantly increased the number of adherent human leukocytes to the human vascular endothelium (Figure 2B). Blocking of either P- or E-selectin or both, however, did not modify leukocyte adhesion pointing to P- and E- selectin independent mediation of L-NAME-triggered leukocyte adhesion.

**Figure 3:**
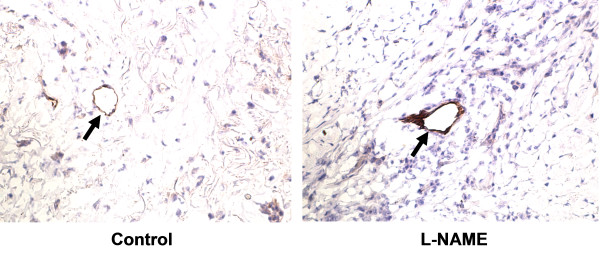
**L-NAME-induced upregulation of E-selectin expression in vivo.** Immunohistochemical analysis (representative of 4 specimens each) showing E-selectin expression (arrow) in human microvasculature of the human skin xenografts in SCID-hu mice without treatment (control, left panel) and 4 h following L-NAME administration (50 mg/kg b.w., i.p., right panel).

Immunohistochemical analysis revealed the increased E-selectin expression in human skin vessels from L-NAME-treated mice in comparison to the human skin in untreated mice (Figure 3) pointing to the regulation of E-selectin expression in human microvasculature by endogenous NO.

**Figure 4:**
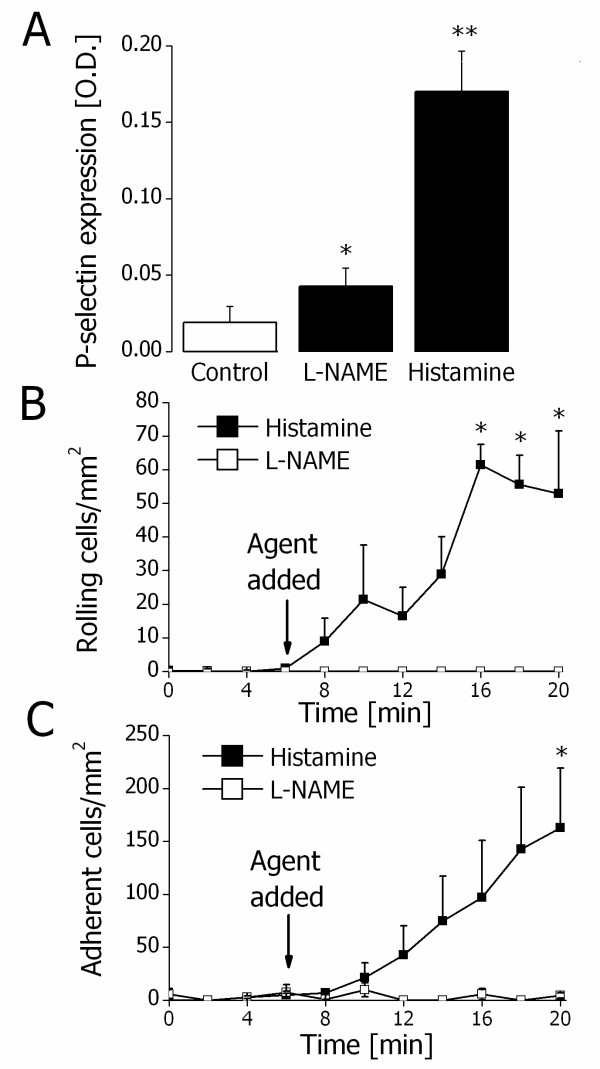
**L-NAME-sensitive P-selectin expression and function in vitro. A**. Endothelial P-selectin expression in controls (white bar) and after treatment with L-NAME (100 μM) or histamine (25 μM) for 10 min (black bars). * and ** indicate P < 0.05 and P < 0.01, respectively, difference from the control (*t* test). Data are means ± SEM (n = 4). **B**. Rolling neutrophils before and after treatment of HUVECs with 100 μM L-NAME (white squares) or 25 μM histamine (black squares). * indicates P < 0.05 difference from the value prior to treatment (ANOVA). Data are means ± SEM (n = 4). **C**. Adherent neutrophils before and after treatment of HUVECs with 100 μM L-NAME (white squares) or 25 μM histamine (black squares). * indicates P < 0.05 difference from the value prior to treatment (ANOVA). Data are means ± SEM (n = 4).

In view of these observations, together with the limitations of complexity and nonspecificity of cellular reactions in vivo, we tested the hypothesis that NO depletion affects P- and E-selectin expression and function in human endothelial cells in vitro. HUVECs treated with L-NAME (100 μM) for 10 min showed a significant increase in the expression of P-selectin in comparison to untreated HUVECs. Histamine treatment (25 μM) also produced a significant increase in P-selectin expression (Figure 4A). Additional experiments explored the effects of increased P-selectin expression on HUVECs on neutrophil rolling or adhesion in an in vitro flow chamber. L-NAME treatment had no effect on the rolling or adhesion of neutrophils over the time points studied, whereas histamine treatment significantly increased neutrophil rolling and adhesion (Figures 4B and 4C).

**Figure 5:**
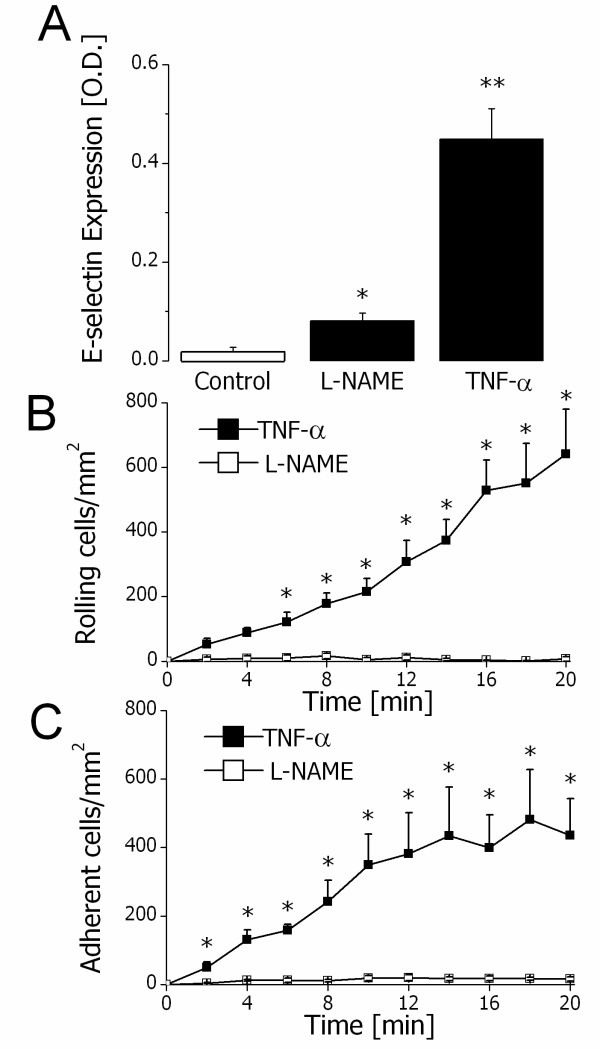
**L-NAME-sensitive E-selectin expression and function in vitro. A**. Endothelial E-selectin expression in controls (white bar) and 4 hours after treatment with L-NAME (100 μM) or TNFα (10 ng/mL) (black bars). * and ** indicate P < 0.05 and P < 0.01, respectively, difference from the control (*t* test). Data are means ± SEM (n = 4). **B**. Rolling neutrophils 4 h after treatment of HUVECs with 100 μM L-NAME (white squares) or 10 ng/ml TNFα (black squares). * indicates P < 0.05 difference from the value at time point 0 min (ANOVA). Data are means ± SEM (n = 4). **C**. Adhesion of neutrophils 4 h after treatment of HUVECs with 100 μM L-NAME (white squares) or 10 ng/mL TNFα (black squares). * indicates P < 0.05 difference from the value at time point 0 min (ANOVA). Data are means ± SEM (n = 4).

A similar approach was taken to analyze the effect of NO synthesis inhibition on E-selectin expression and function. HUVECs treated with L-NAME (100 μM) or TNFα (10 ng/ml) for 4 h showed a significant increase in E-selectin expression in comparison to untreated HUVECs (Figure 5A). Following TNFα treatment, there was a significant increase in neutrophil rolling and adhesion, while L-NAME treatment had no effect (Figures 5B and 5C) pointing to a discrepancy between P- and E-selectin expression and function in vitro.

As PAF may foster adherence of leukocytes on endothelium, additional experiments were performed to investigate the effect of inhibiting endothelial NO on PAF synthesis. Treatment of HUVECs with 100 μM L-NAME did not significantly modify PAF synthesis (229.3 ± 64.0 cpm, n = 3) in comparison to untreated HUVECs (186.6 ± 2.6 cpm, n = 3). Addition of 10 μM histamine to HUVECs significantly augmented PAF synthesis. However, there was no significant difference in histamine-induced PAF synthesis between HUVECs treated with 100 μM L-NAME (3797.3 ± 1157.3 cpm, n = 3) and untreated HUVECs (3360.0 ± 746.6 cpm, n = 3). These results suggest endothelial mechanisms independent of PAF synthesis in L-NAME-modulated early events of rolling and adhesion in leukocyte recruitment.

## Discussion

The present study discloses a novel approach to investigate the mechanism of leukocyte recruitment in human vasculature, a model of using intravital microscopy to directly visualize and determine human leukocyte-endothelial cell interactions in vivo. This method was previously reported only in animal models and in the study of the interactions between *Plasmodium falciparum*-infected human erythrocytes and human endothelium [24]. Studies performed in various in vitro or animal models have shown the role of NO-mediated signaling in leukocyte recruitment [12,14,30-34], but studies on human vasculature have not been reported. Using intravital microscopy, we investigated early recruitment steps of human leukocyte rolling and adhesion in human microvasculature in the SCID-hu mouse model. We show that inhibition of NO synthesis with L-NAME enhances early human leukocyte recruitment steps of rolling and adhesion in vivo. Our results of enhanced rolling and adhesion of human leukocytes on human vessels after inhibiting NO synthesis are consistent with previous studies [14,30,35].

As shown previously, selectins mediate leukocyte rolling but not adhesion [36-41]. In this study we demonstrate that in microvasculature of human skin, functional blocking of P- and E-selectins resulted in a decrease of rolling but not adhesion elicited by L-NAME. The interplay between endogenous NO and endothelial selectin expression remains unclear although previous studies show that inhibition of NO may enhance the expression of endothelial adhesion molecules including selectins [42,43]. E-selectin expression was previously shown to be modulated by L-NAME [44] an effect that may be mediated by NF-κB [45]. The present study further reveals that L-NAME treatment in vitro increased the expression of both P- and E-selectins which, however, did not effect either rolling or adhesion under in vitro flow conditions. We observed subtle but significantly enhanced endothelial P-selectin expression following 10 min of L-NAME treatment. In contrast, prolonged L-NAME treatment was previously shown to trigger P-selectin synthesis rather than its rapid mobilization in endothelial cells as evidenced by increased P-selectin mRNA levels [46,47]. It has previously been reported that L-NAME treatment upregulated P-selectin expression and stimulated neutrophil rolling and adhesion in vitro [21]. However, these observations were made at relatively lower shear rates and on bovine arterial endothelium in contrast to HUVECs used in the present study. Interestingly, NO generation in cultured endothelial [48] and smooth muscle cells [49] may be sensitive to in vitro flow conditions.

The discrepancy between our in vitro and in vivo observations may be attributed to the complexity of cellular interactions and associated mediators in vivo. It is intriguing to speculate the role of mast cells in NO-mediated leukocyte recruitment in vivo as they are in a close proximity to the vasculature and display enhanced sensitivity to pro-inflammatory mediators. Mast cell stabilizers were reported to attenuate L-NAME-induced leukocyte recruitment in vivo [50]. Addition of L-NAME to a co-culture of mast cells and endothelial cells was indeed shown to stimulate leukocyte adhesion [51] which may be mediated by the generation of peroxynitrite [52]. Furthermore, mast cell degranulation was shown to upregulate E-selectin expression and leukocyte recruitment in the SCID-hu mouse model [35]. We observed that endothelial PAF synthesis upon stimulation with L-NAME in vitro was not significantly modified which provides further evidence to the involvement of other players, such as mast cells, during leukocyte recruitment.

## Conclusions

The present observations reveal that pharmacological inhibition of NO synthesis by L-NAME enhances human leukocyte rolling and adhesion in the human microvasculature. L-NAME triggered rolling is mediated by P- and E-selectin expression. Although the increased expression of selectins on isolated HUVECs did not modify the functions of rolling and adhesion in vitro, it strongly suggests the implication of other possible players in L-NAME-mediated human leukocyte recruitment in vivo.

## Abbreviations

NO, Nitric oxide; SCID, severe combined immunodeficiency; L-NAME, NG-nitro-L-arginine methyl ester; ELISA, enzyme-linked immunosorbent assay; TLC, thin layer chromatography; HUVECs, human umbilical vein endothelial cells; PAF, platelet-activating factor; TNFα, tumor necrosis factor-α.

## Competing interests

The authors state that they have no conflict of interest to disclose.

## Authors’ contributions

LL designed the study and conducted the experiments. MH, SMQ and LL analyzed the data and drafted the manuscript. All authors read and approved the manuscript.
